# From metabolic intermediary to neurotransmitter: purinergic signaling in neurophysiology and vulnerability of brain circuits

**DOI:** 10.1042/BSR20250349

**Published:** 2026-08-25

**Authors:** Lucas Bonfim Marques, Allan de Carvalho, Raphael Luiz Lobo da Silva Souza, Fernanda Tibolla Viero, Luiz Roberto G. Britto, Henning Ulrich

**Affiliations:** 1 Department of Biochemistry, Institute of Chemistry, University of São Paulo, São Paulo, SP, Brazil; 2 Department of Biochemistry and Molecular Biology, Federal University of Viçosa, Viçosa, MG, Brazil; 3 Department of Physiology and Biophysics, Institute of Biomedical Sciences, University of São Paulo, São Paulo, SP, Brazil

**Keywords:** brainstem, cerebellum, epileptic seizure, hippocampus, Huntington's disease, neurodegeneration

## Abstract

Purinergic signaling represents an ancient intercellular communication system. Operating at the interface between bioenergetic metabolism and damage detection, extracellular purines such as adenosine 5′-triphosphate (ATP) and adenosine (ADO) are recruited into the nervous system for neurotransmission and homeostatic control. Here, we discuss how purine-dependent mechanisms modulate excitation–inhibition balance, neuroplasticity, and neurodevelopment. In the hippocampus, ADO exerts inhibitory tonic functions, while ATP is released during high neuronal activity, being considered a destabilizing factor during pathological conditions. In the striatum, purinergic signaling intersects with dopaminergic and glutamatergic pathways in the fine-tuning of motor control, with ADO receptors modulating corticostriatal transmission and influencing vulnerability to excitotoxicity and neurodegeneration. Focusing on the cerebellum, we illustrate how purinergic signaling contributes to neurodevelopment and neuroplasticity, and how dysfunctions of the purinergic signaling system give rise to associated neuropathologies. In the brainstem, we demonstrate how purinergic signaling modulates autonomic and respiratory circuits to maintain cardiorespiratory homeostasis through astrocyte–neuron communication. Despite its adaptive value, purinergic signaling is optimized for transient, activity-, or damage-dependent activation. Thus, we argue that, under contemporary conditions characterized by chronic stress and extended lifespan, the purinergic signaling system may undergo a shift from predominantly acute to chronic engagement. This evolutionary mismatch may promote maladaptive purinergic activity, contributing to its involvement in synaptic dysfunction, neuroinflammation, and selective vulnerability of brain circuits. Moreover, we integrate evolutionary, neurophysiological, and neuropathological perspectives to propose purinergic signaling as a key mechanistic bridge linking neural function, bioenergetic metabolism, molecular pathways, and neurodegenerative diseases.

## Introduction

Purinergic signaling appeared early in evolution [1–3]. In addition to purines being essential components of nucleic acids, adenosine 5′-triphosphate (ATP) and adenosine (ADO) have emerged as primary metabolites in bioenergetic pathways [4–8]. However, accumulation of extracellular ATP is interpreted as a damage-associated molecular pattern (DAMP) [3,5,9,10]. For this reason, purinergic signaling is understood as a system that allows the integration of molecular pathways, bioenergetic metabolism, and cell communication [1,4–6].

Glycolysis and oxidative phosphorylation are the main catabolic pathways contributing to intracellular ATP production [1,4,6,11,12]. The hydrolysis of the produced ATP can provide free energy for anabolic reactions and many other cellular processes, including those related to neurotransmission [13,14]. Furthermore, purines participate in the pentose phosphate pathway, and dysfunctions in this pathway can result in pathologies related to the purinergic signaling system [15,16]. The roots of purines, however, are quite ancient, and the function of these molecules traces back to theories concerning their catalytic roles [17].

In eukaryotic cells, intracellular ATP reaches milimolar cytosolic concentrations, while the extracellular ATP concentration is maintained in the nanomolar range [1,3,18]. This concentration gradient favors the efflux of ATP [1,3]. Once secreted into the extracellular space, ATP can directly signal via P2 receptors (P2X and P2Y) or be rapidly degraded by ectonucleotidases into adenosine diphosphate (ADP), adenosine monophosphate (AMP), and ADO, which activates P1 receptors (A_1_, A_2A_, A_2B_, and A_3_), generating a dynamic signaling cascade rather than an isolated ligand–receptor event. Thus, purinergic signaling is shaped not only by receptor expression but also by ectoenzymes and membrane transporters [1,3,19–21].

Intracellular ADO concentrations must also be maintained in equilibrium and, therefore, are balanced by enzymatic activities (e.g., adenosine kinase, ADK; and adenosine deaminase, ADA) and membrane transporters (e.g., equilibrative nucleoside transporters, ENT) [19,22–24]. Extracellular ADO concentrations range from 20 to 300 nM but can reach concentrations of 10 μM in pathological states [20,21,25,26]. Although ADO release from the intracellular environment is relevant, the main source of extracellular ADO is the degradation of ATP by ectonucleotidases [1,11,19,27,28].

This organization gives purinergic signaling a broad temporal and functional range. The coexistence of ionotropic (P2X) and metabotropic receptors (P2Y and P1), extra- and intracellular degrading enzymes, and membrane transporters allow purinergic signaling to operate across phasic and tonic modes, rather than only within a single timescale. These properties are relevant to the nervous system, where metabolic reactions, synaptic activity, and glia-mediated regulation of neuronal functions are tightly coupled [3,19,28–31].

The forebrain offers a particularly informative environment for examining the purinergic signaling system. As vertebrate telencephalic and diencephalic circuits expanded, they supported larger contextual integration, behavioral flexibility, and association processing, but at an increased energetic and computational cost [32–35]. At the same time, circuits characterized by recurrent excitation and strong plasticity require signaling systems that stabilize excitability without eliminating flexibility [36]. Accordingly, the forebrain can be viewed as a progressive elaboration of deeply conserved organizational principles [35].

The hippocampus and striatum provide two circuit models for examining how purines contribute to both the control of homeostasis and circuit failure. In the hippocampus, ATP and ADO dynamics shape neuroplasticity and help control hyperexcitability in temporal lobe epilepsy (TLE) [27]. In the striatum, P1 receptors modulate glutamatergic and dopaminergic neurotransmission and excitotoxicity, making it a pharmacological target for the treatment of Huntington’s disease (HD) and Parkinson’s disease (PD) [25,37]. Furthermore, the primordial function of extracellular ATP and ADP remains in the central nervous system (CNS) as an alert signal capable of promoting and modulating neuroinflammation, in both acute and chronic neurological conditions [3,19,38–42].

Beyond the forebrain, the cerebellum has emerged as a highly organized and functionally specialized structure that extends far beyond its classical role in motor coordination [43,44]. Recent studies demonstrate that the cerebellum exhibits hierarchical, modular, and small-world network architectures, with bilateral Crus I and II acting as major connectivity hubs within cerebro-cerebellar circuits [45]. Functional atlases further reveal distinct motor, cognitive, social, and spatial domains organized into primary, secondary, and tertiary representations that are differentially coupled to cerebral cortical networks, enabling precise sensorimotor integration as well as higher-order cognitive processing [46]. The posterior cerebellum displays a hierarchical functional gradient that parallels the prefrontal cortex, supporting progressively abstract cognitive control from motor-proximal to future-oriented behavioral planning [47]. Consistent with this organization, cerebellar circuits contribute to flexible behavior, associative learning, fear memory, and social recognition through extensive interactions with the thalamus, hypothalamus, prefrontal cortex, hippocampus, amygdala, and brainstem nuclei [48–51]. At the developmental level, single-cell multiomic analyses have identified hierarchical transcriptional programs and human-specific molecular regulators governing cerebellar neurogenesis, neuronal differentiation, and circuit assembly, while disease-associated genetic variants map to distinct cerebellar cell populations implicated in neuropsychiatric disorders [52].

Similarly, large-scale functional connectivity analyses demonstrate that brainstem nuclei form highly interconnected communities spanning the midbrain, pons, and medulla, exhibiting stronger functional coupling with the cerebral cortex than among themselves and organizing cortical networks involved in memory, emotion regulation, sensory perception, and cognitive control [53]. Genetic studies further support the importance of these structures by identifying the first loci specifically associated with midbrain, pontine, and medullary morphology, including developmental regulators such as *HOX* genes and *LMX1A*, while revealing significant genetic overlap with schizophrenia, bipolar disorder, PD, and multiple sclerosis [54]. Recent electrophysiological studies also demonstrate that brainstem circuits actively encode threat probability, prediction error, rapid eye movement (REM) sleep regulation, and behavioral state transitions through functionally specialized neuronal networks [55,56].

Collectively, these findings establish the cerebellum and brainstem as integral components of distributed brain circuits, suggesting that purinergic signaling may play important roles in modulating brain development, connectivity, physiological function, and pathological remodeling.

In this review, we first examine how nucleotide-derived molecules acquired the properties of intercellular chemical transmitters and neurotransmitters. We then discuss how the purinergic signaling system regulates excitability in hippocampal and striatal networks. We also present the purinergic system roles in cerebellum and brainstem development and function. Finally, we propose that prolonged exposure to metabolic, inflammatory, and psychological stressors, combined with extended lifespan, promotes a shift from predominantly acute to sustained purinergic engagement, providing a conceptual link between neurophysiological signaling and its dysregulation in brain disorders.

## From metabolic intermediary to neurotransmitter: how nucleotide-derived molecules became brain neurotransmitters

### Ancient metabolic and evolutionary roots of purinergic signaling

The biochemical properties of ATP and nucleotide-derived molecules made them suitable extracellular signaling molecules, and evolutionary processes favored cellular mechanisms capable of detecting and responding to these purines, contributing to the emergence of one of the earliest chemical signaling systems [2,5,6].

Consistent with the early emergence of ATP as a chemical signaling molecule, trimeric ionotropic P2X receptors emerged early in eukaryotic evolution, whereas metabotropic purinergic receptors appear to have evolved later [1,3,5,57–61]. The selection of both classes of metabotropic purinergic receptors would only be possible in later stages of the evolutionary process, with P1 receptors for ADO appearing first, followed by the P2Y receptors for ATP and other nucleotides [5]. Finally, and almost as old as the first purinergic receptors, was the emergence of ATP-degrading enzymes, generically known as ectonucleotidases [1].

This early recruitment likely reflected function as much as phylogeny. Purinergic pathways are tightly linked to membrane excitability, calcium handling, and cellular integrity [3,9,10,62]. Extracellular ATP can signal cellular activity, stress, or injury, whereas its conversion to ADO often shifts the local purinergic environment toward restraining and homeostatic modulation. This capacity to alter the functional meaning of the signal over time gives purinergic signaling a homeostatic logic that is unusual among neurotransmitter systems and may have contributed to its broad evolutionary conservation. In this view, purinergic signaling was not merely retained because it was ancient, but because its structure made it especially useful for coupling cellular conditions to extracellular communication.

### Recruitment of purines into neurotransmission

The nervous system is present only in Metazoa and likely appeared first within the basal epitheliozoan lineage (Cnidaria, Ctenophora, and Bilateria), excluding Porifera [63–66]. Both ctenophores and cnidarians possess diffuse nerve nets, while bilaterians exhibit a centralized nervous system [65,66]. In early-diverging animals, neurons communicate through chemical synapses, specifically through ionotropic channels [5]. In the invertebrate *Aplysia californica* (Mollusca), for example, ATP-gated P2X receptors are expressed in an integrative neurosecretory center [67]. Although other representative organisms of this group, such as the arthropod *Drosophila melanogaster* and the nematode *Caenorhabditis elegans*, have lost genes encoding P2X receptors during evolution, they retain genes encoding ectonucleotidases [67,68].

The evolutionary selection of purinergic ligands into systems of neurotransmission may have been favored by their ubiquity in cellular metabolism [62]. Like any other neurotransmission system, the purinergic signaling system possesses the molecular infrastructure necessary for the synthesis, transport, release, and degradation of purines [1,28,69,70]. Furthermore, the coexistence of multiple classes of purinergic receptors and purine-converting enzymes enables purinergic signaling to operate across different temporal and spatial scales [28,71–73].

Taken together, several intrinsic characteristics likely promoted the evolutionary incorporation of purines into neurotransmission. These properties allowed purinergic signaling to function simultaneously as a neurotransmitter, co-neurotransmitter, and neuromodulator, making it particularly well-suited for homeostatic control and integration into the expanding brain circuitry of vertebrates.

## Purinergic signaling in the hippocampus

From an evolutionary perspective, the hippocampus represents a highly conserved structure across vertebrates, with homologous circuits supporting spatial and associative memory functions despite variations in cytoarchitecture across taxa [74]. Comparative analyses indicate that, although the morphology of the hippocampus differs among reptiles, birds, and mammals, its functions appear to be evolutionarily preserved. The hippocampal formation consists of the association between the hippocampus, dentate gyrus, and subiculum [75–77]. This structure is mainly made up of glutamatergic pyramidal and granular neurons (excitatory) and GABAergic interneurons (inhibitory). These cells present inputs with different neurochemical properties and are part of the trisynaptic circuit. This circuit begins from the synapse between the pyramidal neurons of the medial entorhinal cortex (medial entorhinal cortex – MEC) and the granule neurons of the dentate gyrus (synapse 1). The latter projects toward the CA3 pyramidal neurons (synapse 2 – mossy fiber pathway), which are afferent to the CA1 pyramidal neurons of the hippocampus (synapse 3 – Schaffer collateral). Hippocampal CA1 pyramidal neurons develop long-term potentiation (LTP) and long-term depression (LTD) responses, which represent a neuroplasticity phenomenon correlated to the processing, formation, storage, and retrieval functions of declarative and spatial memories [76–78]. The ionotropic NMDA (*N*-methyl-D-aspartate), AMPA (α-amino-3-hydroxy-5-methyl-4-isoxazolepropionate), and KA (kainic acid) glutamatergic receptors are involved in LTP formation and excitotoxic phenomena through the influx of extracellular Ca^2+^ [76,79]. High-frequency stimulations of NMDA receptors result in Ca^2+^ influx followed by activation of a Ca^2+^/calmodulin-dependent protein kinase cascade [76]. In contrast, prolonged low-frequency stimulations result in Ca^2+^ influx followed by the activation of a cascade of phosphatases [76]. Furthermore, the hippocampus is the region most affected in TLE, a pathophysiology conceived as an imbalance between excitatory and inhibitory activity within a neuronal circuit due to abnormal and increased synchronous neuronal activation [80,81]. Characteristic pathological findings of temporal lobe epilepsies include hippocampal sclerosis, resulting from loss of excitatory and inhibitory neurons. The pathophysiological mechanisms of epilepsy are mainly explained by abnormal activities of cortical neurons. However, glial cells also affect neuronal networks [82,83]. This way, neuromodulatory and anticonvulsant therapies can consider both synaptic and heterosynaptic neural communication. In the case of epilepsy, pharmacological treatments are mainly based on reducing neuronal excitation and increasing neuronal inhibition [84]. In this scenario, both ATP and ADO have neuromodulatory effects on hippocampal neurons [11,27]. ADO appears as an endogenous anticonvulsant and neuroprotective neuromodulator of the brain [85]. ATP, on the other hand, acts as a pro-convulsant neurotransmitter [27,86–88].

### Purinergic signaling in hippocampal neuromodulation and neuroplasticity

**Figure 1:**
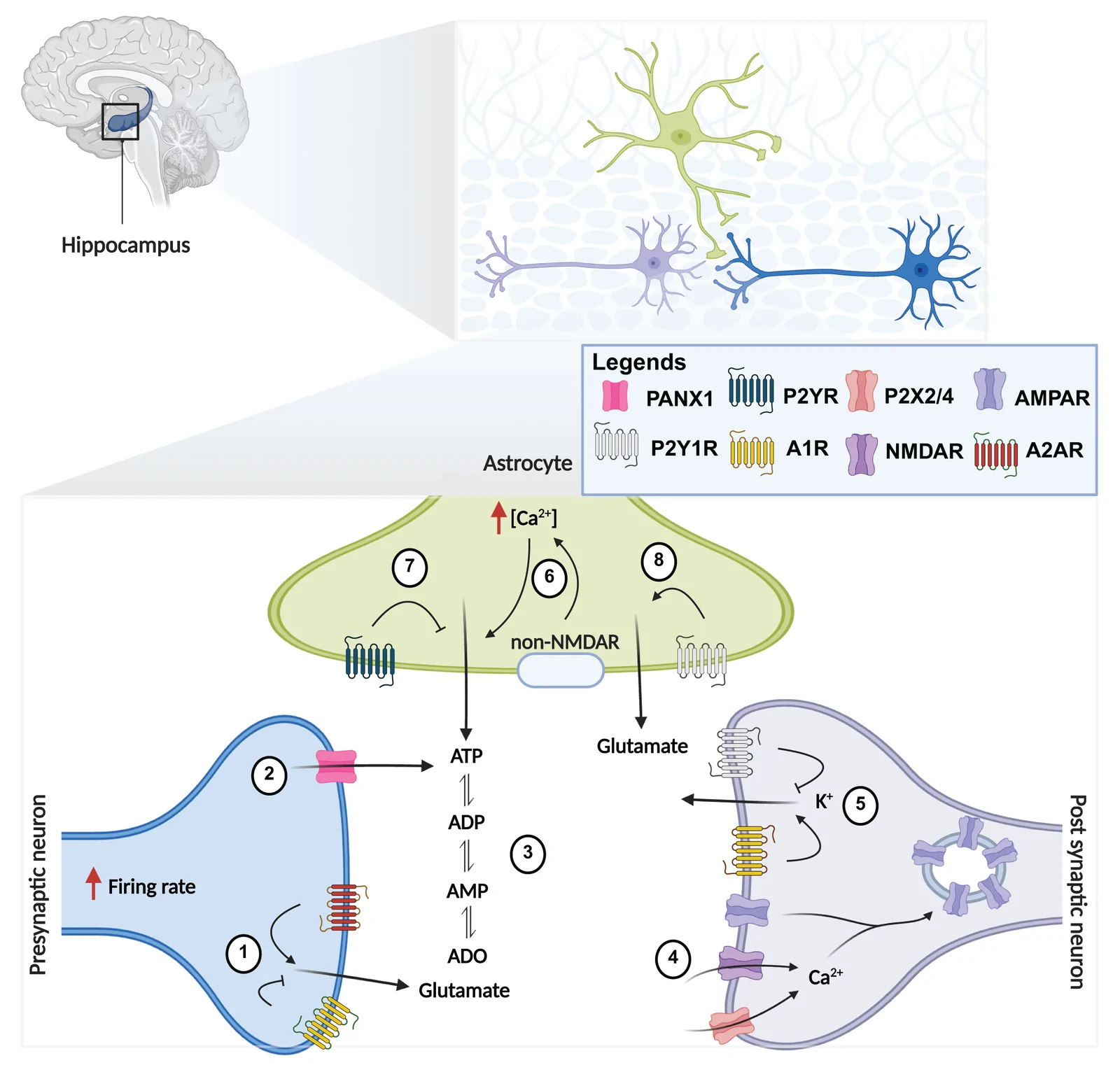
Neuromodulation by purinergic signaling in the hippocampus 1, Extracellular ADO regulates glutamate release by activating the inhibitory A_1_ receptor and the excitatory A_2A_ receptor. 2, During states of neural hyperexcitability, ATP is released through PANX1 channels. 3, Production of ADO from ATP through the activities of ectonucleotidases. 4, By activating P2X2/4 receptors, ATP decreases NMDA receptor responses and promotes AMPA receptor internalization. 5, Neuromodulation of the postsynaptic membrane potential through stimulation or inhibition of K+ channels. 6, The increase in [Ca^2+^]_i_ through the activation of non-NMDA receptors in astrocytes promotes the release of glutamate. 7, Inhibition of ATP release by astrocytic P2Y receptors. 8, ATP promotes the release of glutamate by activating P2Y1 receptors on astrocytes.

In the CA1 region of the hippocampus, under physiological conditions, extracellular ATP activates P2Y1 receptors and leads to increased neuronal excitability through the inhibition of two-pore potassium channels (Figure 1) [89]. This effect leads to neuronal network stimulation when P2Y1 receptors are activated on excitatory neurons, or to inhibition when they are activated on inhibitory neurons [89,90]. On the other hand, ATP can also activate P2X receptors in the CA1 region and reduce neuronal excitability through a calcium-dependent mechanism of AMPA receptor internalization [11,91,92]. In this sense, blocking P2X4 receptors modulates NMDA receptor activity in a way that favors LTP [93,94].

The hydrolysis of extracellular ATP into ADO by the activity of ectonucleotidases results in the activation of P1 receptors [1,11,28,40]. Thus, activation of presynaptic neuronal A_1_ receptors by ADO inhibits glutamate release and, therefore, decreases the excitability of postsynaptic neurons [89,95]. ADO can also reduce postsynaptic neuronal excitability by activating the G_i/o_-coupled A_1_ receptor, which promotes hyperpolarization through the activation of G-protein-coupled inwardly rectifying potassium channels [96]. Conversely, activation of presynaptic neuronal G_s_-coupled A_2A_ receptors stimulates the release of glutamate and, consequently, increases the excitation of postsynaptic neurons [97]. Thus, since A_1_ receptors have a higher affinity for ADO than A_2A_ receptors do, the purinergic system contributes to the regulation of the homeostatic excitation–inhibition balance through the activation of A_1_ and A_2A_ receptors, which inhibit and stimulate glutamate release into the synaptic cleft, respectively [98].

In astrocytes, the activation of glutamatergic receptors increases [Ca^2+^]_i_, resulting in the release of ATP into the synaptic cleft via exocytosis [11,99]. On the other hand, the activation of their P2Y receptors by ATP inhibits the release of glutamate [99]. In addition, ATP can be released by astrocytes through the pannexin-1 (PANX-1) channel, as well as through the opening of the P2X7 receptor pore [89,100–102]. Following ATP hydrolysis, extracellular ADO can decrease synaptic activity via A_1_ receptor activation [103]. However, an unbalanced high extracellular concentration of ADO that exceeds the levels associated with the inhibitory effects of A_1_ receptors can also activate A_2A_ receptors, resulting in glutamate release [104,105].

Among purinergic mechanisms in the hippocampus, tonic activation of A_1_ receptors plays a central modulatory role [106]. Electrophysiological recordings in hippocampal slices demonstrate the presence of an endogenous ADO tone that continuously modulates synaptic transmission [107]. Unlike classical phasic neuromodulators released in response to discrete events, extracellular ADO is present under basal conditions and exerts a persistent inhibitory influence on excitatory synapses [108]. This basal ADO tone shapes the operating range of synaptic transmission by limiting excessive excitation while preserving responsiveness to patterned inputs [109,110].

Accordingly, basal extracellular ADO levels are sufficient to sustain tonic A_1_ receptor activation, resulting in continuous inhibition of excitatory synapses [110,111]. As described, A_1_ receptors implement this control through coordinated pre- and post-synaptic mechanisms, reducing the likelihood of glutamate release and promoting membrane hyperpolarization, respectively [112–114]. Together, these effects reduce synaptic gain and stabilize network activity within a constrained functional range [110,111,115]. Thus, activity-dependent ADO accumulation suppresses excessive excitatory transmission in hippocampal slices [116].

ADO acting through A_1_ receptors regulates synaptic plasticity by modulating induction thresholds rather than silencing transmission [117]. Because LTP depends on postsynaptic Ca^2+^ influx through NMDA receptors and voltage-dependent channels, A_1_ receptor-mediated control of membrane excitability directly influences the threshold for LTP induction [118]. Consistently, endogenous ADO suppresses LTP induction without abolishing basal synaptic transmission [119]. Under certain stimulation conditions, reduced excitability may instead favor LTD, indicating that ADO can influence the direction of synaptic plasticity [120].

In parallel, ATP-mediated signaling provides a complementary and dynamically coupled mechanism. When ATP release exceeds local degradation capacity, or when ectonucleotidase activity is altered, P2 receptor-mediated excitation may transiently predominate before sufficient ADO-mediated inhibition has been established [121]. Thus, ATP/P2 and ADO/P1 receptor signaling cascades form an integrated regulatory axis, whose net effect depends on receptor subtype expression, spatial distribution, extracellular nucleotide dynamics, and metabolic states [121].

Dysregulation of extracellular ATP levels, however, is associated with neuropathological conditions [11]. Elevated ATP concentrations can activate low-affinity receptors such as the P2X7 subtypes [11,122]. Because the P2X7 receptor requires high ATP levels for activation, it is generally considered a sensor of intense activity or pathological states rather than a constitutive modulator of synaptic transmission [31]. In hippocampal models, P2X7 receptor activation has been associated with neuroinflammatory signaling, increased neuronal excitability, and enhanced seizure susceptibility [123]. Together, these findings indicate that ATP/P2 receptor signaling can shift from a modulatory to a destabilizing role under conditions of sustained or excessive ATP accumulation.

### Purinergic neuromodulation in temporal lobe epileptic seizures

Epilepsy is a group of disorders characterized by a predisposition to the occurrence of epileptic seizures, the causes of which are distinct [81,124]. The etiology of epilepsy includes genetic, structural, and metabolic causes, and is associated with lesions, tumors, inflammation, and ion channel dysfunction [125,126]. The models used to study them can be genetic, structural, electrical, or chemical [125–127]. Genetic models may be based on selection of traits that predispose the animal to epileptic seizures by increasing sensitivity to external stimuli or disrupting the excitation–inhibition balance. These models can also be based on the promotion of tumorigenesis, which leads to cell proliferation and predisposes the animal to seizures by causing sclerosis, a mechanism like that of structural or lesion-induced models, which promote sclerosis by causing neurodegeneration. Electrical models, in turn, rely on repeated subthreshold electrical stimulation that triggers seizures and are used to study seizure sensitization. Chemical models of epilepsy act on glutamate, GABA, or muscarinic receptors, for example, disrupting the balance between neuronal excitation and inhibition [126,128,129]. Finally, it is important to consider that sclerosis is a common denominator that predominates among the temporal lobe epilepsies [130–132].

Epileptic seizure is defined as “a transient occurrence of signs and/or symptoms due to abnormal excessive or synchronous neuronal activity in the brain” [82]. Epileptogenesis is the process resulting from an imbalance between excitatory and inhibitory activity within the neuronal network [82]. *Status epilepticus*, in turn, is a condition of prolonged and recurrent seizures [124]. In mouse models, it is characterized on the EEG by a continuous spike-wave pattern lasting at least 3 h [133].

TLE is a general term for a pathology that includes the most common focal subtypes of epilepsy, with mesial temporal lobe epilepsy (mTLE) affecting the circuit integrated by the hippocampus, entorhinal cortex, amygdala, and parahippocampal gyrus [80]. Experimental models of TLE consistently identify hippocampus as a key site of epileptogenesis [134,135]. In this subtopic, we analyze the functions of neuronal and astrocytic P1 and P2 receptors, the roles of purine transporters and purine-converting enzymes, and the influence of bioenergetic metabolism on ATP and ADO activities during epileptic seizures in the hippocampus.

Neuronal activity controls intracellular ATP concentrations, while its activity is regulated by extracellular ATP [11,136]. These mechanisms of regulation integrate the metabolic pathways and, therefore, the cellular states of strong or low energy demand also influence neuronal functions. Thus, extracellular ATP acts as a neuromodulator that regulates intracellular homeostatic processes [11]. During neuronal activation, the intracellular concentration of ATP increases, which is followed by an increase in the extracellular concentration of ATP through its release by exocytosis via synaptic vesicles in nerve terminals or by diffusion through volume-gated anion channels located in the axons [11].

During epileptic seizures, pannexin (e.g., PANX1) channels appear to be involved in ATP release and, consequently, in P2 receptor activation [137–139]. While the origin of epileptogenesis is not always known, epileptic seizures develop following an alteration in neuronal excitability, such as the synchronization of neural activity, or a loss of excitation–inhibition control in the neural network [137,139]. After this period, pannexin channels are activated, resulting in the release of ATP into the extracellular environment and the activation of P2 receptors. While activation of these channels promotes seizures, their inhibition and genetic knockout have anticonvulsant effects. ATP activates P2 receptors, while its degradation product ADO activates P1 receptors [140]. In general, P2 receptors have been associated with the excitation of the neuronal network, while P1 receptors promote its inhibition. Therefore, pannexin channels have been linked to the maintenance of epileptic seizures through the activation of P2 receptors by ATP, but also to the anticonvulsant effects of P1 receptors activated by ATP-derived ADO. This mechanism is observed in the kainic acid (KA)-induced *status epilepticus* model [140].

Due to its rapid degradation by ectonucleotidases, the extracellular ATP concentration will reach its maximum level after its release into the extracellular environment. Thus, the low-affinity P2X7 receptor will be activated in the acute phase of the epileptic seizure, being associated with its exacerbation, and considered a marker of these states [1,141–143]. However, P2X7 receptors are primarily expressed by microglia [86], and antagonists of these receptors have little effect against epileptic seizures [144]. Thus, extracellular ATP appears to potentiate epileptic seizures primarily through the activation of P2Y1 receptors in astrocytes by ATP and ADP, inducing a Ca^2+^-dependent glutamate release, although it can also occur through the activation of P2X receptors, contributing to neuronal depolarization [27,86–88,139]. The P2Y12 receptor, in turn, acts in epilepsy through the interaction between microglia and neurons [145]. In the kainic acid (KA)-induced epileptic seizure, activation of the microglia P2Y12 receptor appears to increase the hyperexcitability of the neuronal network [146,147]. Therefore, the epileptogenic effects of ATP may also be associated with neuroinflammation [148]. In this context, P2X4 receptors participate in microglia activation, which is linked to increased neurodegeneration [149].

A counterpoint to the hypothesis that ATP can promote seizures by rapidly increasing neuronal excitability is the evidence that the major fast P2X receptors, P2X2 and P2X4, are not expressed near presynaptic terminals [11]. Thus, extracellular ATP is believed to be a neuromodulator that reduces hyperexcitability by depolarizing inhibitory interneurons through activation of the P2Y1 receptor, rather than stimulating CA1 excitatory pyramidal neurons [31]. Another point of controversy stems from the difficulty of measuring extracellular ATP because of their low concentrations and rapid degradation by ectonucleotidases [27,141].

Extracellular ADO produced from ATP by ectonucleotidases activates postsynaptic A_1_ receptors, promoting hyperpolarization, inhibits glutamate release through presynaptic A_1_ receptors, and stimulates glutamate release through presynaptic A_2A_ receptors [71,104,150]. Thus, A_1_ receptor signaling is strongly implicated in seizure suppression and modulation of epileptogenesis, reinforcing its potential role as a stabilizing influence in amplification-sensitive circuits [133,151,152]. Taken together, available evidence supports the interpretation that hippocampal purinergic signaling, particularly tonic A_1_ receptor activation, may function as a constitutive regulatory constraint embedded within recurrent memory networks, coupling excitability, plasticity thresholds, and metabolic state [4,86,153,154]. However, excessively high concentrations of ADO can activate the proconvulsant A_2A_ receptors [155]. Furthermore, these antagonistic effects of ADO depend on the kinetics of P1 receptors. The A_1_ receptor exhibits slow internalization and desensitization profiles, while the A_2A_ receptor exhibits rapid profiles [156]. Therefore, the kinetic profiles of P1 receptors also favor the anticonvulsant effects of ADO.

Astrocytes possess several mechanisms that lead to the release of ATP into the extracellular environment, with increased intracellular free calcium concentration ([Ca^2+^]_i_) being a common denominator [11,89,90,95,97]. Extracellular ATP released by astrocytes does not only activate P2 neuronal receptors, but its hydrolysis into ADO also leads to the activation of P1 neuronal receptors [19,86]. Furthermore, astrocytes strongly express the enzyme ADK, which plays a central role in maintaining the excitation–inhibition homeostatic control through the clearance of ADO [153,155,157]. Since ADO has been initially associated with inhibitory effects and increased ADK expression with decreased ADO concentrations, increased ADK expression has been correlated with a higher likelihood of seizures [152,157–159]. ADK has low capacity and high affinity for ADO [103]. These properties make this enzyme a rate-limiting factor under physiological conditions. The enzyme adenosine deaminase (ADA), on the other hand, has a high capacity and a low affinity, which makes this enzyme functional at high intracellular ADO concentrations. These enzymes interconnect metabolism and synaptic activity [4,103]. For this reason, ADK inhibition has been proposed as a therapeutic target to increase ADO signaling [103,154].

**Figure 2:**
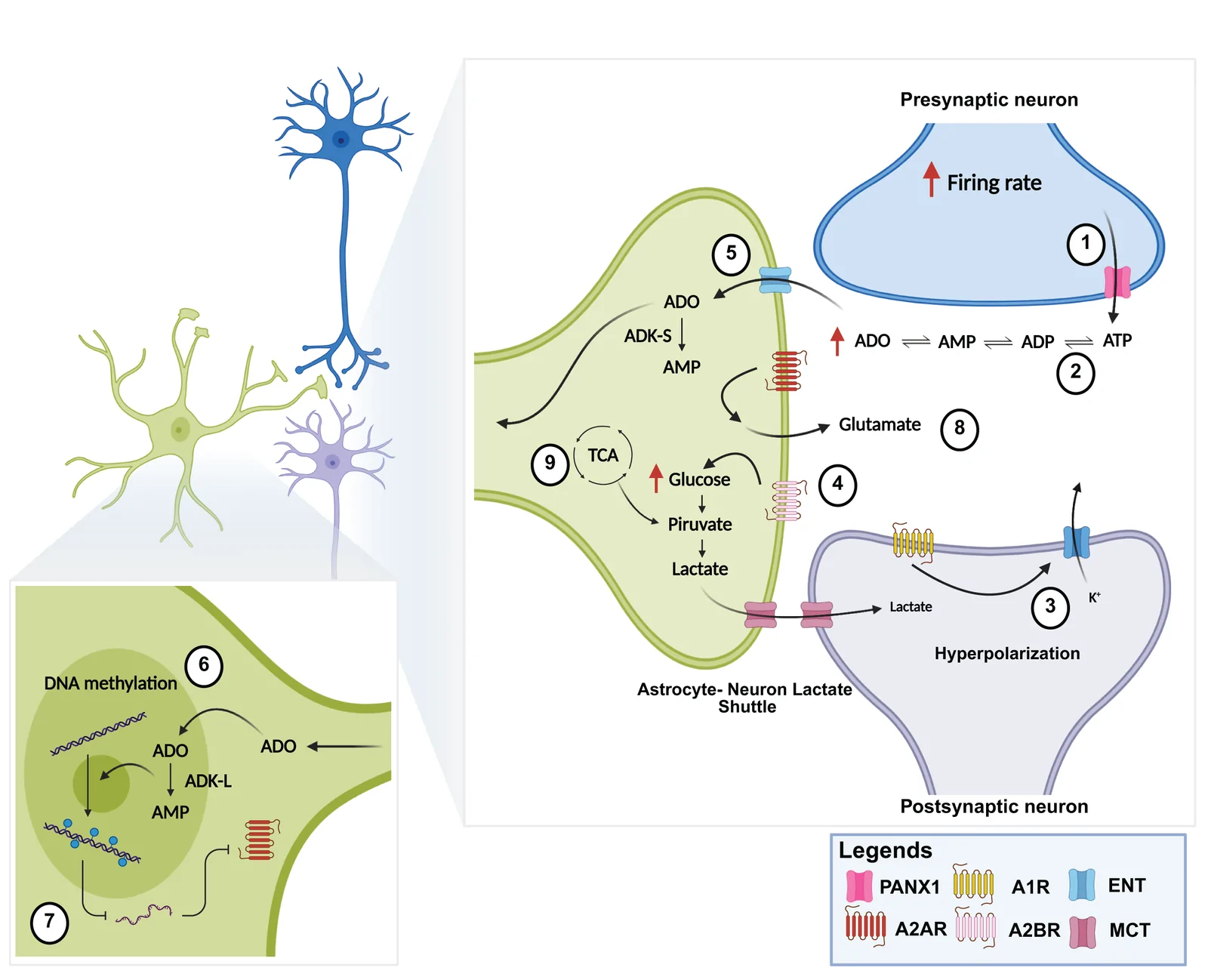
Integration of purinergic neurotransmission with bioenergetic metabolism in the control of epileptic seizures 1, During states of neural hyperexcitability, ATP is released through PANX1 channels. 2, Production of ADO from ATP through the activities of ectonucleotidases. 3, ADO activates A_1_ receptors, promoting hyperpolarization of postsynaptic neurons through the activation of G-protein-coupled inwardly rectifying potassium channels. 4, Activation of A_2B_ receptors in astrocytes stimulates glucose metabolism and lactate production, which is transferred to neurons via the astrocyte–neuron lactate shuttle. 5, ADO transport via ENTs. 6, In the nucleus, the ADO phosphorylation reaction catalyzed by ADK-L increases the rate of DNA methylation. 7, Increased DNA methylation inhibits the expression of A_2A_ receptors. 8, Activation of A_2A_ receptors stimulates the release of glutamate. 9, Ketogenic diets promote lactate production and recovery from epileptic seizures.

The integration of the purinergic system into metabolic and molecular pathways in the face of the reestablishment of homeostatic control can be exemplified by the fact that increased ADO levels, followed by increased ADK activity and ADO deficiency, has been correlated with the mechanism of DNA methylation, while DNA methylation is inversely related to A_2A_ receptor expression (Figure 2) [4,154,160]. There are two main isoforms of ADK, one found in the cytoplasm, called ADK-S, and another found in the nucleus, called ADK-L [4]. The role of bioenergetic metabolism in these pathways can be exemplified in epileptic seizures by the fact that ketogenic diets increase ADO levels, which activate A_1_ receptors, promoting anticonvulsant effects, as well as facilitating epileptic seizure recovery [4,86,154]. Bioenergetic metabolism is complemented by astrocytic A_2B_ receptors, whose activation stimulates glucose metabolism in astrocytes and lactate release in the brain. Activation of astrocytic metabolism depends on neuronal activity and is part of the metabolic coupling mechanism between neurons and astrocytes (astrocyte–neuron lactate shuttle) via monocarboxylic acid transporters (MCTs). In situations of high energy demand and low energy supply, the activation of A_2B_ receptors in astrocytes by ADO provides neurons with alternative sources of energy [161].

Thus, as described, we note that, during epileptic seizures, the purinergic system functions in an integrated manner with bioenergetic metabolism and gene regulation, with all its components (receptors, transporters, and enzymes) working to restore homeostasis.

## Purinergic signaling in the striatum: neurophysiology and neuropathology

The basal ganglia (BG) are composed of a set of interconnected subcortical anatomical structures [162,163]. These structures are subclassified according to the regions around the longitudinal axis of the CNS. In the dorsal sector, we find the neostriatum (caudate and putamen), globus pallidus (external, GPe; and internal, GPi), substantia nigra (SN), and subthalamic nucleus (STN). Neurons in the motor cortex synapse with neurons in the dorsal portion to coordinate voluntary body movements [164,165].

**Figure 3:**
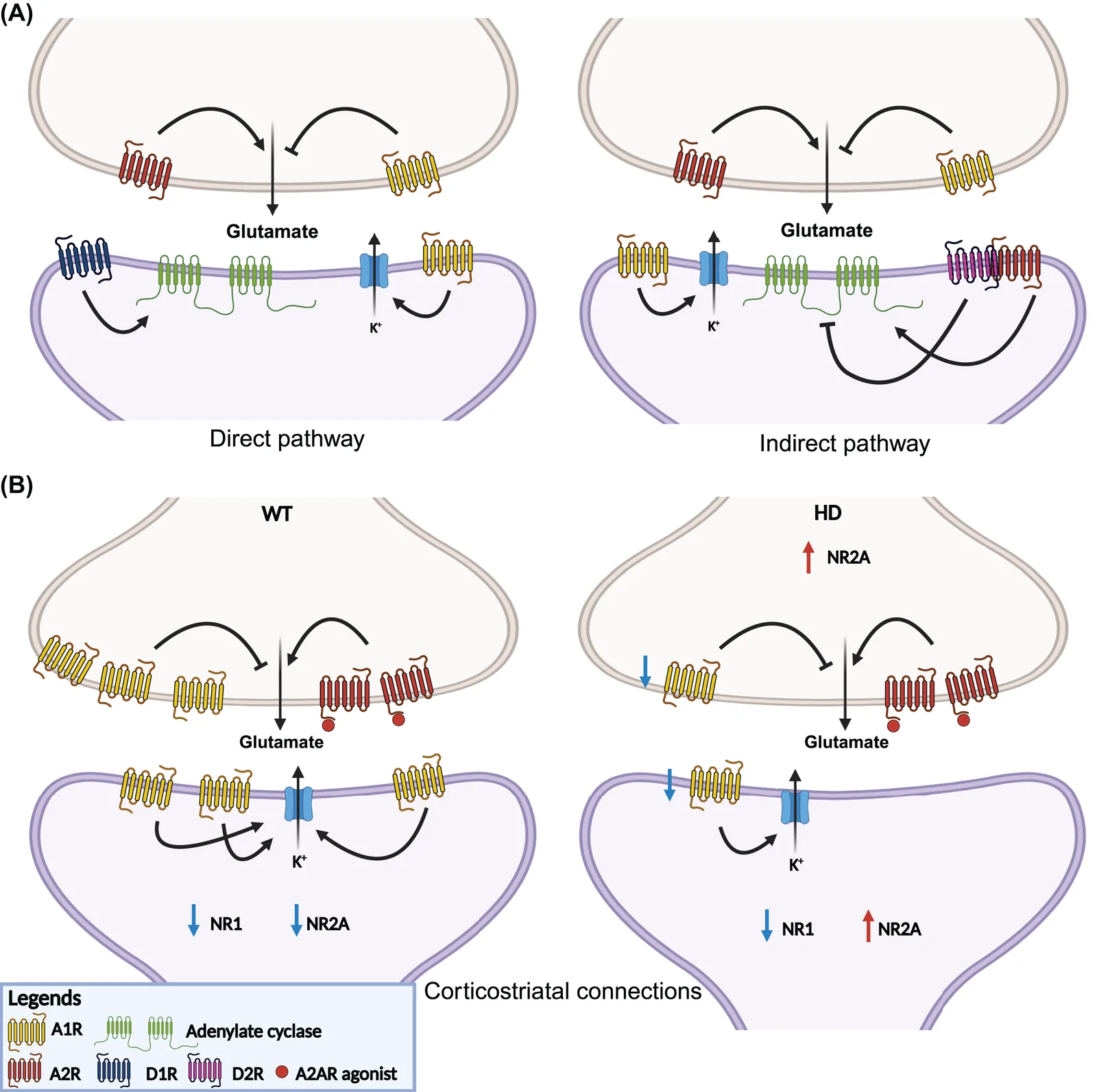
Purinergic neuromodulation in the striatum (rats) (**A**) Direct and indirect pathways. ADO exerts inhibitory effects by activating A_1_ receptors and stimulatory effects by activating A_2A_ receptors, which regulate glutamate release through the presynaptic terminals of glutamatergic pyramidal neurons in the cortex. In the postsynaptic neurons, A_1_ receptors promote hyperpolarization through the activation of inwardly rectifying potassium channels. In the direct pathway, D1 receptors coupled to the Gs protein activate AC. In the indirect pathway, D2 receptors coupled to the Gi protein inhibit AC. However, in the indirect pathway, D2 receptors are co-located with Gs-coupled A_2A_ receptors. (**B**) Neuromodulation of corticostriatal connections by an A_2A_ receptor agonist (mouse). In this picture, there is no distinction between the direct and indirect pathways. In wild-type (WT) mice (left side), increased glutamate release induces a response of reduced expression of NMDA receptor subunits by postsynaptic neurons. In HD mice, in which there is a significant loss of corticostriatal connections in advanced stages, a reduction in the expression of inhibitory A_1_ receptors is observed, as well as an increase in the expression of the NR2A subunit and a decrease in the NR1 subunit. This picture reflects that the release of glutamate induced by the A_2A_ receptor agonist in WT mice results in an adjustment of the degree of excitability of the postsynaptic neuron in a way that reduces responsiveness to glutamate, while in rHD mice there is a tendency toward increased neuronal excitability due to decreased expression of the inhibitory A_1_ receptor, as well as increased responsiveness to glutamate due to increased expression of NMDA receptor subunits, probably due to the opposite function of the A_2A_ receptor in advanced stages of HD, where the presence of an A_2A_ receptor agonist inhibits glutamate release.

The synapses formed between glutamatergic pyramidal neurons of the cortex and GABAergic medium spiny neurons (MSNs) of the striatum are known as corticostriatal connections [162,164–167]. The cortico–BG circuit coordinates motor functions predominantly through two pathways called direct (striatonigral) and indirect (striatopallidal) (Figure 3A) [164,166,168].

Both the direct and indirect pathways originate in the cortical glutamatergic pyramidal neurons that project toward the GABAergic MSNs of the striatum [162,165,167,169]. These GABAergic MSNs of the striatum are characterized by the differential expression of dopamine receptors [170]. D1-positive GABAergic MSNs integrate the direct pathway, which is related to the stimulatory functions of motor activity, while D2-positive GABAergic MSNs integrate the indirect pathway, which is related to the inhibitory functions of motor activity [171].

Both D1-positive and D2-positive GABAergic MSNs receive afferents from dopaminergic neurons in the substantia nigra pars compacta (SNpc) via the nigrostriatal pathway [165,172]. D1 receptors are excitatory, while D2 receptors are inhibitory [171,172]. These effects occur through the increase and decrease in cyclic adenosine monophosphate (cAMP) production from the stimulation and inhibition of adenylate cyclase (AC), which enhances or represses CaV1 (L) calcium currents, respectively [171,173]. Thus, dopamine promotes the facilitation of body movements by stimulating D1-positive MSNs in the direct pathway, as well as by inhibiting D2-positive MSNs in the indirect pathway [164,173]. Furthermore, dopamine influences NMDAR-dependent neuronal plasticity by modulating cAMP production [164,172]. In this sense, NMDA receptors play a role in establishing dopamine-dependent behavioral functions [174].

In this topic, we explore how the purinergic signaling system neuromodulates striatal functions related to glutamatergic and dopaminergic signaling, based on findings obtained by studies on HD and PD. Since A_2A_ receptors are predominantly expressed in the striatum [175], with elevated levels in neurological diseases and an inverse relationship with DNA methylation [160], they are the main focus of our discussion.

### Direct pathway

The direct pathway originates in the D1-positive GABAergic MSNs of the striatum, which project efferent fibers toward the GABAergic neurons of the GPi and the substantia nigra pars reticulata (SNr) via the striatonigral pathway [162,165,167]. When the GABAergic neurons of the GPi/SNr are inhibited by signals from the GABAergic MSNs of the striatum, the former ceases to inhibit the glutamatergic thalamic pyramidal neurons. Consequently, glutamatergic thalamic pyramidal neurons release higher amounts of glutamate in their thalamocortical connections with the cortical pyramidal neurons, which results in glutamate release in the corticostriatal connections. The *de novo* stimulation of GABAergic MSNs in the striatum via the direct pathway represents a positive feedback mechanism [162,165]. The neurophysiological outcome of the direct pathway is the facilitation of body movements [162,165,176]. Furthermore, activation of excitatory D1 receptors via the nigrostriatal pathway stimulates MSNs in the direct pathway, promoting the facilitation of body movements [164,173].

In the corticostriatal connections that integrate the direct pathway, presynaptic glutamatergic pyramidal neurons in the cortex express both A_1_ and A_2A_ receptors on their synaptic terminals, while postsynaptic D1-positive GABAergic MSNs express only A_1_ receptors on their dendrites [25]. Thus, ADO inhibits glutamate release by activating presynaptic A_1_ receptors, while stimulating glutamate release by activating presynaptic A_2A_ receptors [25,177,178]. Therefore, activation of the presynaptic A_1_ receptor reduces synaptic transmission [177]. On the other hand, ADO promotes neuronal hyperpolarization through the activation of postsynaptic A_1_ receptors [25].

### Indirect pathway

The indirect pathway originates in the D2-positive GABAergic MSNs of the striatum, which project terminals toward the GABAergic neurons of the GPe via the striatopallidal pathway [162,165,167]. When inhibited, GABAergic neurons in the GPe cease to inhibit glutamatergic neurons in the STN. Disinhibited, subthalamic glutamatergic neurons stimulate the GPi/SNr GABAergic neurons. Thus, excitatory stimulation of GPi/SNr GABAergic neurons results in increased inhibition of pyramidal glutamatergic neurons in the thalamus, which decreases glutamate release at thalamocortical connections. Consequently, unstimulated cortical pyramidal glutamatergic neurons decrease glutamate release at corticostriatal connections. Therefore, the reduction of cortical stimuli through the activation of GABAergic MSNs in the striatum, via an indirect pathway, is a negative feedback mechanism, which promotes the reduction of motor activity [162,164,165,176]. However, the facilitation of body movements can be favored by the inhibition of GABAergic D2-positive MSNs through activation of their D2 receptors by dopaminergic neurons in the SNpc, decreasing activity via the indirect pathway [164,173].

In the corticostriatal connections that integrate the indirect pathway, presynaptic glutamatergic pyramidal neurons in the cortex express A_1_ and A_2A_ receptors at their synaptic terminals, while postsynaptic D2-positive GABAergic MSNs, in addition to expressing A_1_ receptors, express the A_2A_ receptor co-localized with the D2 receptor [25,179,180]. In the same way, ADO inhibits glutamate release by activating presynaptic A_1_ receptors, while stimulating glutamate release by activating presynaptic A_2A_ receptors [25,177,178]. On the other hand, activation of G_s_ protein-coupled A_2A_ receptors by ADO antagonizes the effects of activation of G_I_/G_o_-coupled D2 receptors by dopamine, in addition to decreasing their affinity for the ligand [180,181]. Consequently, the activation of A_2A_ receptors stimulates cAMP production, increases the activity of the striatopallidal pathway, and results in the inhibition of body movements [179,180].

In patients with PD, the A_2A_ receptor antagonist istradefylline has been co-administered with l-DOPA to potentiate the effects of the latter in controlling motor dysfunction without causing dyskinesia [172]. In patients with HD, on the other hand, it is observed that the GABAergic D2-positive MSNs are the most affected by neurodegenerative processes, which is reflected in the motor disorder characterized by hyperkinesia [182]. One of the main causes of neurodegeneration of GABAergic D2-positive MSNs is NMDAR-dependent excitotoxicity [182,183]. Lastly, it is known that low concentrations of ADO or alterations in the expression or function of A_2A_ receptors, accompanied by dysfunctions in heterodimerization with D2 receptors and hyperdopaminergic states, have been associated with other neuropathologies, such as schizophrenia [184].

The role of adenosine receptors in the overstimulation of NMDARs was analyzed under different conditions using the HD model based on intrastriatal injection of quinolinic acid (QA). In this sense, activation of A_1_ receptors inhibits presynaptic glutamate release in the R6/2 transgenic mouse model of HD and, consequently, promotes neuroprotection against excitotoxicity [185]. Furthermore, the expression of A_1_ receptors is reduced in R6/2 mice when compared to that of WT mice [185]. On the other hand, inhibition of A_2A_ receptors protects against QA-induced excitotoxicity through a presynaptic mechanism [186,187]. This effect is attributed to the inhibition of presynaptic glutamate release mediated by the A_2A_ receptor [186]. However, when the WT mouse is compared with the R6/2 transgenic mouse model of HD, treatments with the A_2A_ receptor agonist have dissimilar effects, potentiating NMDAR toxicity in the WT mouse but not in the R6/2 mouse [188]. One possible hypothesis to explain this result is the loss of corticostriatal connections that integrate the indirect pathway in the R6/2 mouse, the main entity subjected to the effects of the A_2A_ receptor agonist in potentiating NMDAR-induced excitotoxicity.

The distinct pre- and post-synaptic effects of treatment with the A_2A_ receptor antagonist have already been demonstrated, showing that the anti-excitotoxic effects of the A_2A_ receptor antagonist are attributed to the inhibition of presynaptic glutamate release [189]. On the other hand, overexpression of the A_2A_ receptor is linked to neuroprotection, while its reduced expression is observed during pathologies [190,191]. This effect is explained by two mechanisms: BDNF production and modulation of the expression of NMDA receptor subunits [189,192,193].

The postsynaptic effect of A_2A_ receptors related to excitotoxicity and alteration of the expression of NMDAR subunits was analyzed in WT and R6/2 mice chronically treated with the A_2A_ receptor agonist [194]. In the cortex, the A_2A_ receptor agonist induced an increase in NR2A expression and in the NR2A/NR2B expression ratio in R6/2 mice but did not promote any changes in WT mice. In the striatum, however, the A_2A_ receptor agonist reduced NR1 expression in both WT and R6/2 mice, while it reduced and increased NR2 expression and the NR2/NR2B ratio in WT and R6/2 mice, respectively. Analysis of the excitotoxicity results showed no difference between the models. NMDARs are composed of four subunits, two NR1 and two NR2 (A–D) [194]. NR1 subunits bind to glycine, while NR2 subunits bind to glutamate [195]. The NR2B subunit is associated with increased susceptibility to excitotoxicity [196].

Therefore, we can infer that purinergic signaling plays an essential role in controlling synaptic tone in the corticostriatal connections, both by altering the expression of purinergic receptors and by modulating the expression of NMDA receptor subunits (Figure 3B). In the HD models, characterized by neurodegeneration of excitatory glutamatergic corticostriatal connections and alterations in the intrinsic properties of the membrane and synapses [197,198], it was demonstrated that there is lower expression of A_1_ receptors and higher expression of NMDA receptor subunits that are more sensitive to glutamate action, probably due to decreased glutamate release in the later stages of this neuropathology [198]. In HD murine models, the explanation of the effects of A_2A_ receptor activation and blockade on glutamatergic signaling is complex and remains under investigation, with evidence of distinct pre- and post-synaptic mechanisms and responses opposite to those noted in WT models, such as increased glutamate release through treatment with A_2A_ receptor antagonists [188,189,194,199]. On the other hand, in the WT mouse, activation of A_2A_ receptors and their consequent facilitation of glutamate release reduced the expression of NMDA receptor subunits, thus decreasing responsiveness to glutamate, which would now be present in higher concentrations in the synaptic cleft.

### P2 receptors in striatal neuromodulation and neuroinflammation

The role of ATP signaling in the striatum is not yet well understood. Concerning neuromodulation, ATP produces inhibitory effects on glutamatergic signaling [200]. Although the mechanism remains uncertain, the maximal inhibitory effect of ATP, greater than that of P1 receptor activation by ADO, illustrates both the role of ATP as a striatal inhibitory neuromodulator and its importance as a source of ADO for the control of synaptic tone. On the other hand, regarding neuroinflammation, the most described P2 receptors are P2X4 and P2X7 subtypes. Expression levels of the P2X4 receptor are inversely related to extracellular ATP concentration and associated with the intensification of inflammatory processes [201]. Although the P2X4 receptor is also expressed by GABAergic striatal neurons, in the context of neuroinflammation, ATP production was related to microglial activation by the proinflammatory mediator IL-1β and, consequently, microglial activation was associated with decreased expression of the P2X4 receptor. The other ATP-binding receptor, the low-affinity P2X7 receptor, which is also expressed in the striatum during pathological states, primarily contributes to neuroinflammation, and has been observed in both HD and PD [177,202,203].

## Purinergic signaling in cerebellar and brainstem circuits

Not only forebrain circuits intrinsically depend on purinergic signaling but also brain structures in hindbrain and midbrain regions, such as the cerebellum and the brainstem. Although traditionally associated with motor coordination, the cerebellum is increasingly recognized as an important hub for cognitive, behavioral, and neuropsychiatric functions [49,204–206], with purinergic signaling emerging as an important regulator of its development, plasticity, and response to pathological stimuli [207–211].

Unlike most forebrain regions, ADK-L remains highly expressed in cerebellar granule neurons and Bergmann glia throughout adulthood, while it is the exclusive ADK isoform in granule neuron precursors during development, where its inhibition reduces DNA synthesis and impairs cell proliferation [208]. Consistent with these findings, genetic deletion of ADK-L and ADK-S overexpression leads to profound cerebellar abnormalities, including reduced cerebellar volume, altered foliation, impaired granule cell proliferation and migration, increased apoptosis, and defective Purkinje cell dendritic arborization [211]. Together, these observations identify ADK-L as a critical mediator of adenosine-dependent metabolic and epigenetic mechanisms that coordinate cerebellar growth, neuronal differentiation, and long-term plasticity [208,211].

Chronic gestational and lactational glutamate exposure disrupts cerebellar purinergic signaling by reducing A_1_ receptor expression, thereby potentially weakening the inhibitory control of ADO over glutamatergic neurotransmission and contributing to increased GluR1 expression, oxidative stress, and heightened susceptibility to excitotoxic damage [209]. In parallel, ATP-mediated activation of P2X7 receptor regulates cell survival pathways in cerebellar astrocytes and granule neurons through the induction of the MAPK phosphatase DUSP1, a process mediated by ERK1/2-, p38-, EGFR-, and BDNF-dependent signaling cascades in a cell type-specific manner [210].

Metabolomic and transcriptomic approaches demonstrated that the response to nicotine withdrawal is accompanied by upregulation of A_2A_ receptor signaling and down-regulation of A_1_ receptor and A_3_ receptor in the cerebellum, shifting ADO signaling toward activation of the cAMP–PKA and neuronal nitric oxide synthase-dependent nitric oxide–cGMP pathways [207,212]. These molecular alterations are associated with anxiety- and depression-like behaviors, while pharmacological blockade of adenosine receptors with theobromine attenuates behavioral symptoms and ADO signaling, and restores disrupted arginine metabolism [207].

The brainstem is now recognized as a central hub of neural integration, linking autonomic and respiratory control with large-scale cortical networks involved in cognition, emotion, and motor function [53]. Recent evidence further indicates that the structural and genetic organization of specific brainstem regions contributes to the pathophysiology of several neurological and psychiatric disorders [54]. Within these circuits, recent evidence indicated that purinergic signaling plays a key role in regulating vascular tone, chemoreception, respiratory rhythmogenesis, and cardiorespiratory reflex processing [213–216].

In the rostral ventrolateral medulla, activation of P2Y1 receptor increases vascular tone and supports sympathetic activity, contributing to chronic hypoperfusion, elevated arterial pressure, and exaggerated chemoreflex responses in hypertension, whereas receptor blockade reduces vessel constriction, sympathetic outflow, and cardiovascular activation [213]. In the nucleus tractus solitarius, purinergic signaling cooperates with glutamatergic neurotransmission to mediate the autonomic and respiratory components of the peripheral chemoreflex, as combined nonselective blockade of P2 and glutamate receptors markedly suppresses chemoreflex-evoked responses [214]. Astrocytes further modulate these pathways by regulating parasympathetic and respiratory outputs, although their influence is altered under sustained hypoxia [214].

In the retrotrapezoid nucleus, the astrocyte-derived ATP signaling through P2X7 receptor is essential for maintaining regular breathing, and chronic heart failure disrupts this pathway by reducing astrocytic activity, ATP bioavailability, and P2X7 receptor expression, leading to respiratory instability and periodic breathing that can be reversed by restoring astrocytic purinergic signaling [215]. In the preBötzinger Complex, astrocytic [Ca^2+^]_i_ elevations triggered by stimuli such as hypoxia or β-adrenergic activation promote ATP release and ADP-mediated activation of P2Y1 receptors on neurons and astrocytes, selectively driving spontaneous and hypoxia-induced sigh generation without substantially affecting eupneic breathing [216].

## Evolutionary mismatch in the purinergic signaling control of brain circuits

### From acute to chronic activation of purinergic signaling in the contemporary brain

As discussed in the first section of this review, ATP acts as both an energetic resource and an extracellular signal. Earlier in evolution, the nucleotide gradient between intra- and extracellular environment favored the role of ATP as an immediate indicator of integrity disruption and damage [217,218]. Burnstock and Verkhratsky emphasized that purinergic signaling is one of the most ancient intercellular communication systems and operates across prokaryotes, protozoa, and higher eukaryotes, underscoring its basal role in alarm signaling [1].

This initial framework, centered on rapid detection and localized response, has been refined by three properties. First, there are multiple release pathways from both living, injured or dying cells [72,219]. Second, receptor families have distinct kinetics and coupling mechanisms, enhancing the system complexity with temporal and functional diversity [220,221]. Third, ectonucleotidases establish a cascade in which the degradation products are themselves biologically active signals [222].

Together, these features confer an acute design for purinergic signaling: ATP release is episodic and activity- or damage-dependent, whereas its removal is rapid and enzymatically organized, preventing sustained receptor occupancy [223,224]. The notion that the ATP degradation system is conserved further reinforces that rapid signal termination is intrinsic to the system’s design [225]. However, ATP and ADO do not only act as episodic damage sensors in the human brain [226,227] but also as modulators of the network state and homeostasis [161,228].

In the brain, purinergic signaling has been recruited for functions that surpass its primitive alarm logic. ATP is now recognized as a cotransmitter [229,230] or the sole neurotransmitter [231], being widely distributed throughout the CNS. It exerts both rapid (e.g. upon cellular excitability) and long-term, or trophic, effects on cell proliferation, growth, development, and cytotoxicity, as recently reviewed by our group [232–235].

These long-term effects upon homeostasis and network state require a sustained function of the purinergic signaling, referred here as chronic activation. The shift from acute to chronic activation may be understood as a mismatch between a system evolutionarily tuned for discrete events and the current environment characterized by repeated, cumulative, and relatively low-intensity but persistent stimuli [236,237]. Current urban lifestyle presents unprecedented scenarios of social isolation, poor housing and working environments, as well as physical inactivity, which are translated into higher stress levels within human organisms [238]. Rapid lifestyle changes also promote increasing levels of psychological stressors [239].

Consistent levels of stressful stimuli recruit recurrent purinergic activation through the purinergic–inflammasome axis in brain structures [240,241]. As recently reported, psychological stress was linked to inflammatory stimulation via ATP and the P2X7 receptor, with increased interleukin-1β levels and activation of the NLRP3 inflammasome, as well as reversal of stress-induced behavioral phenotypes following P2X7 receptor antagonism or *Nlrp3* deletion in mice [242,243].

Regarding the adenosinergic pathway, subchronic stress may reduce A_1_ receptor expression while markedly increasing A_2A_ receptor immunoreactivity in the hippocampus, with synaptic preservation observed following A_2A_ antagonism [244]. Similar shifts in A_1_ versus A_2A_ receptor expression and sensitivity have been reported in models of chronic unpredictable stress, where A_2A_ receptor overexpression is associated with depressive-like behavior and impaired stress resilience [245,246].

### Chronic metabolic load in the center of selective vulnerability of brain circuitry

Contemporary life is accompanied by recurrent exposure and incomplete recovery from stressors, energetic challenges, and inflammatory signals [247–249]. In the brain, this translates into sustained engagement of intrinsically energy-demanding circuits, such as in the hippocampus [250], prefrontal cortex [251,252], and striatum [253]. Resulting metabolic state changes can become maladaptive when chronically sustained [254–256].

Acute stress rapidly recruits norepinephrine and glucocorticoids, influencing hippocampal-dependent memory processes [257–259]. These signals dynamically shape substrate utilization. Prolonged glucocorticoid elevation has been associated with reduced glucose metabolism, excitotoxic vulnerability, and memory impairments [260,261].

Brain neurons operate with minimal energy reserves and depend on continuous delivery of oxygen and metabolic substrates [262]. They remain engaged in high-rate computational functions, whereas glial cells, especially astrocytes, support substrate uptake, buffer metabolic fluctuations through glycogen stores, and rapidly adjust supply to activity (Figure 2) [161,263,264].

Together, these findings indicate that chronic stress or prolonged energetic challenge leads to sustained high extracellular ATP and ADO levels. Persistent activation of purinergic receptors promotes synaptic dysfunction and behavioral alterations [72,265,266]. Consequently, contemporary conditions are indirectly linked to impaired synaptic plasticity and the selective vulnerability of brain circuits, which become more likely to the development of neuropsychiatric and neurodegenerative disorders [267–269].

### The impact of the lifespan extension upon purinergic signaling modulation

The evolutionary mismatch framework explains how biological systems optimized for ancestral environments may become vulnerable under rapidly changing conditions [270]. The dramatic increase in human life expectancy over the past century has led to an unprecedented expansion of late adulthood, a period characterized by the progressive accumulation of molecular damage, chronic low-grade inflammation, and network-level homeostatic strain [271,272].

Within the brain, aging is associated with diverse cellular and molecular alterations, including increased DNA damage, loss of proteostasis, mitochondrial dysfunction, and a shift toward a pro-inflammatory milieu [273–275]. In the hippocampal and cortical circuits, microglia adopt reactive phenotypes and synaptic environments become less permissive to plasticity and repair [273,274].

These events are directly linked to purinergic signaling, as ATP and ADO act as mediators of cellular stress sensing, glial activation, metabolic coupling, and synaptic regulation [161,276,277]. Progressive mitochondrial dysfunction also disrupts ATP availability, reinforcing a feedback loop between metabolic stress and purinergic imbalance [275,278,279].

Evidence from aging model mice showed that A_1_ receptor density declines with age in cortical regions while A_2A_ receptor density increases, with less pronounced changes in the striatum [280]. In humans, PET imaging confirmed age-related reduction of A_1_ receptor levels in the cerebral cortex [281], whereas A_2A_ receptor expression is up-regulated in the aged brain and further enhanced in Alzheimer’s disease [282]. This balance culminates in enhanced adenosinergic inhibition [283].

Astrocytes exhibit reduced ATP-mediated Ca^2+^ signaling driven by P2X1 and P2Y1 receptors and diminished nucleotide release with aging [284], weakening tripartite synapse modulation [285]. Aged microglia also present down-regulation of key nucleotide-sensing receptors such as P2Y12 and P2Y13, impairing their responsiveness and motility [286].

The abovementioned evidence indicates that purinergic signaling is particularly vulnerable under extended lifespan. The onset of a chronic low-grade inflammation environment, currently known as inflammaging [287], places sustained demand on purinergic pathways, which act as amplifiers and regulators of cytokine signaling [227]. The decline in regenerative (e.g. adult neurogenesis) [288] and synaptic plasticity [275,283] resulting from aging also limits the capacity of brain circuits to compensate for persistent stress.

Together, these observations support the idea that purinergic signaling is progressively returned during aging. Mechanisms that evolved to coordinate acute responses are instead chronically engaged over decades. Under these conditions, purinergic signaling modulation may shift from a homeostatic regulator to a contributor of network vulnerability, providing a mechanistic bridge between normal aging and neurodegenerative processes, supported by the evolutionary mismatch framework.

## Conclusion

In this review, purinergic signaling stands out as an ancestral system and a fundamental link between bioenergetic metabolism, molecular pathways, and neurotransmission. In the hippocampus and striatum, we noted that ATP and ADO do not have antagonistic effects. Conversely, activation of P1 and P2 receptors produces responses that depend on the receptor subtype and cell type [1,5,98]. ATP promotes not only neuromodulation but also neuroinflammation [11,31,89–93,122]. ADO, in turn, exhibits both inhibitory and stimulatory effects [89,95–97]. During epileptic seizures, ATP-derived ADO activates anticonvulsant A_1_ receptors, whereas at high concentrations it also activates proconvulsant A_2A_ receptors [155]. Dysregulation of the A_2A_ receptor promotes hyperexcitability and excitotoxicity in both the hippocampus and the striatum [186,289]. In the hippocampus, the A_1_ receptor exerts a central modulatory role, while in the striatum, the A_2A_ receptor predominates [106,172,175,186,187,194]. The integration of the purinergic system with neural biophysics, bioenergetic metabolism, and molecular pathways in restoring homeostasis has been exemplified by the roles of ADO concentration, ADK activity, DNA methylation, A_2A_ receptor expression, astrocytic A_2B_ receptor activation, glucose and lactate production, and ketogenic diets [4,86,154,160]. Furthermore, as illustrated in the cerebellum, the purinergic signaling system also plays regulatory roles in neurodevelopmental processes, influencing neuronal differentiation, neuroplasticity, and adaptive responses to pathological stimuli, by coordinating neuron–glia communication and contributing to behavioral dysfunction when disrupted [207,208,211]. In the brainstem, in turn, it orchestrates cardiovascular and respiratory homeostasis by regulating sympathetic outflow, chemoreflex integration and respiratory rhythm generation through astrocyte–neuron communication [213–216]. Taken together, this review highlights that purinergic signaling, originally adapted for rapid and transient responses to acute cellular stress, is increasingly recruited in a sustained manner in the brain. We discussed how chronic stress, metabolic challenges, and aging reshape ATP and ADO signaling, leading to receptor expression and activity imbalances, altered synaptic function, and neuroinflammatory states, while also reducing the flexibility of purinergic regulation across the lifespan. Importantly, this synthesis advances the concept of an evolutionary mismatch, in which a system optimized for acute activation becomes maladaptively engaged under persistent conditions, providing a unifying framework to understand how purinergic signaling may bridge physiological regulation and vulnerability to brain disorders.

## Declaration on the use of Generative Artificial Intelligence

During the preparation of this manuscript, generative artificial intelligence tools, including ChatGPT, were used exclusively to assist with language refinement, textual cohesion, coherence, grammar correction, and structural organization of the text. These tools were not used to generate scientific interpretations, perform data analysis, select references, or formulate the conclusions of the review. All scientific arguments, interpretations, references, and final textual decisions were critically reviewed, verified, and approved by the authors, who take full responsibility for the integrity, accuracy, and originality of the manuscript.

## Abbreviations

AC: adenylate cyclase
ADA: adenosine deaminase
ADK: adenosine kinase
ADO: adenosine
ADP: adenosine diphosphate
AMP: adenosine monophosphate
AMPA: α-amino-3-hydroxy-5-methyl-4-isoxazolepropionate
ATP: adenosine 5′-triphosphate
BDNF: brain-derived neurotrophic factor
BG: basal ganglia
cAMP: cyclic adenosine monophosphate
cGMP: cyclic guanosine monophosphate
CNS: central nervous system
DAMP: damage-associated molecular pattern
DUSP1: dual-specificity phosphatase 1
EEG: electroencephalogram
EGFR: epidermal growth factor receptor
ENT: equilibrative nucleoside transporter
ERK1/2: extracellular signal-regulated kinases 1 and 2
GABA: gamma-aminobutyric acid
GPe: external globus pallidus
GPi: internal globus pallidus
HD: Huntington’s disease
IL-1β: interleukin-1 beta
KA: kainic acid
L-DOPA: L-3,4-dihydroxyphenylalanine
LTD: long-term depression
LTP: long-term potentiation
MAPK: mitogen-activated protein kinase
MCT: monocarboxylic acid transporter
MEC: medial entorhinal cortex
MSN: medium spiny neuron
mTLE: mesial temporal lobe epilepsy
NLRP3: NLR family pyrin domain containing 3
NMDA: N-methyl-D-aspartate
PANX1: pannexin-1
PD: Parkinson’s disease
PET: positron emission tomography
PKA: protein kinase A
QA: quinolinic acid
REM: rapid eye movement
SN: substantia nigra
SNpc: substantia nigra pars compacta
SNr: substantia nigra pars reticulata
STN: subthalamic nucleus
TLE: temporal lobe epilepsy
WT: wild type
